
JOURNAL INFORMATION
==============================
NLM Title Abbreviation: ScientificWorldJournal
Journal ID: ScientificWorldJournal
NLM Journal ID: 101131163
Journal ID: TSWJ

The Scientific World Journal

ISSN: 2356-6140
EISSN: 1537-744X
Publisher: Wiley

ARTICLE INFORMATION
==============================
PMCID: PMC6083956
DOI: 10.1100/tsw.2001.412
Article ID: 154013
Article version: 1
Subjects: Extended Abstract

Relative mRNA Quantification of Calcitonin Receptor-Like Receptor (CRLR) and Receptor Activity Modifying Proteins (RAMPs) by Real-Time PCR

Eskesen K. 1
Hasbak P. 1
Arendrup H. 2
Edvinsson L. 1
1 Department of Clinical Experimental Research, University Hospital of Copenhagen, Glostrup, Denmark
2 Department of Thoracic Surgery, Rigshospitalet, Copenhagen, Denmark
Publication date: 2001
Electronic publication date: 2001 Dec 8
Volume: 1
First page: 40
Last page: 40
Copyright: Copyright © 2001 K. Eskesen et al.
Copyright year: 2001
License: This is an open access article distributed under the Creative Commons Attribution License, which permits unrestricted use, distribution, and reproduction in any medium, provided the original work is properly cited.
License URL: https://creativecommons.org/licenses/by/3.0/

==============================
4th International Meeting on Calcitonin Gene-Related Peptide (CGRP)
*TheScientificWorld* (2001) 1(S1), 40
ISSN 1532-2246; DOI 10.1100/tsw.2001.412

### RELATIVE mRNA QUANTIFICATION OF CALCITONIN RECEPTOR-LIKE RECEPTOR (CRLR) AND RECEPTOR ACTIVITY MODIFYING PROTEINS (RAMPs) BY REAL-TIME PCR

K. Eskesen, P. Hasbak, H. Arendrup* and L. Edvinsson
Department of Clinical Experimental Research, University Hospital of Copenhagen, Glostrup, Denmark and *Department of Thoracic Surgery, Rigshospitalet, Copenhagen, Denmark

Functional calcitonin gene-related peptide and adrenomedullin receptors appear to consist of CRLR and RAMPs. Three types of human RAMPs have been cloned: RAMP1, RAMP2, and RAMP3.

The aim of this study was to validate the use of real-time polymerase chain reaction (real-time PCR) with regard to evaluation of the relative expression of mRNA for CRLR and RAMPs.

With real-time PCR the input levels of mRNA are correlated to the number of PCR cycles at which the fluorescent emission, here SYBR-green, increases beyond a threshold value. The expression of mRNA of the receptors was quantified relative to β-actin. Sets of primers were designed for CRLR, RAMP1, RAMP2, and RAMP3. For ß-actin we used commercially available primers. The concentration of each primer was optimized and each primer set was tested for efficiency selecting primers that gave a slope of the standard curve close to –3.3. The relative expression of the mRNAs was calculated from the equation: Relative expression = $2^{-\Delta Ct}$, where $\Delta C_t = C_t(\text{sample}) - C_t(\beta\text{-actin})$ and $C_t$ the number of PCR cycles required to reach the chosen threshold value.

With the primers used it was found that in the atrium, ventricles and coronary arteries of the human heart mRNA were present in the following order of abundance: CRLR~RAMP1~RAMP2>RAMP3.

In summary, a real-time PCR method for comparison of the relative expression of mRNA for CRLR and RAMPs in human tissue was validated. Primarily mRNA for CRLR, RAMP1, and RAMP2 are present in the human heart.
